# PtNPs/Short MWCNT-PEDOT: PSS-Modified Microelectrode Array to Detect Neuronal Firing Patterns in the Dorsal Raphe Nucleus and Hippocampus of Insomnia Rats

**DOI:** 10.3390/mi13030488

**Published:** 2022-03-21

**Authors:** Yun Wang, Mixia Wang, Yuchuan Dai, Yilin Song, Yiding Wang, Botao Lu, Yinghui Li, Xinxia Cai

**Affiliations:** 1 State Key Laboratory of Transducer Technology, Aerospace Information Research Institute, Chinese Academy of Sciences, Beijing 100190, China; wangyun17@mails.ucas.ac.cn (Y.W.); wangmixia@mail.ie.ac.cn (M.W.); daiyuchuan18@mails.ucas.ac.cn (Y.D.); ylsong@mail.ie.ac.cn (Y.S.); wangyiding18@mails.ucas.ac.cn (Y.W.); lubotao18@mails.ucas.ac.cn (B.L.); 2 School of Electronic, Electrical and Communication Engineering, University of Chinese Academy of Sciences, Beijing 100049, China; 3 The State Key Laboratory of Space Medicine Fundamentals and Application, China Astronaut Research and Training Center, Beijing 100094, China

**Keywords:** microelectrode array, insomnia, electrophysiology, dorsal raphe nucleus, serotonin

## Abstract

Research on the intracerebral mechanism of insomnia induced by serotonin (5-HT) deficiency is indispensable. In order to explore the effect of 5-HT deficiency-induced insomnia on brain regions related to memory in rats, we designed and fabricated a microelectrode array that simultaneously detects the electrical activity of the dorsal raphe nucleus (DRN) and hippocampus in normal, insomnia and recovery rats in vivo. In the DRN and hippocampus of insomnia rats, our results showed that the spike amplitudes decreased by 40.16 and 57.92%, the spike repolarization slope decreased by 44.64 and 48.59%, and the spiking rate increased by 66.81 and 63.40%. On a mesoscopic scale, the increased firing rates of individual neurons led to an increased δ wave power. In the DRN and hippocampus of insomnia rats, the δ wave power increased by 57.57 and 67.75%. Furthermore, two segments’ δ wave slopes were also increased in two brain regions of the insomnia rats. Our findings suggest that 5-HT deficiency causes the hyperactivity of neurons in the hippocampus and DRN; the DRN’s firing rate and the hippocampal neuronal amplitude reflect insomnia in rats more effectively. Further studies on alleviating neurons affected by 5-HT deficiency and on achieving a highly effective treatment for insomnia by the microelectrode array are needed.

## 1. Introduction

Sleep is a common physiological rhythm phenomenon in humans and higher animals to maintain the body’s health and normal functioning of the central nervous system [1]. There is growing evidence that people worldwide are experiencing a downward trend in the average sleep duration, increasing the prevalence of insomnia and other sleep disorders [2,3,4]. Severe/extreme nocturnal sleep problems are reported by more than 40% of older adults in low-income settings [5]. There is a strong link between insomnia and illness, especially sleep deprivation, which can greatly impair memory and cognitive functions in the body [6,7,8].

The International Classification of Sleep Disorders (ICSD) specifies 11 subtypes of insomnia: acute, psychophysiological, paradoxical, idiopathic, and substance-induced insomnia [9]. The insomnia mechanism is still poorly understood, and abnormal neural activity and neurotransmitter secretion in corresponding brain regions are the main causes of insomnia [10]. Serotonin (5-HT) is an important neurotransmitter, and a lack of 5-HT in the brain is considered a major cause of insomnia [11,12]. Serotonergic neurons are widely distributed in the brain. About two-thirds of the cells in the dorsal raphe nucleus (DRN) of adult rats are serotonergic neurons [13], projecting axons to most of the brain areas, including the cerebral cortex, hypothalamus, amygdala, and hippocampus [14]. The hippocampus is the biological brain region responsible for processing memory and cognition [15,16,17]. Insomnia negatively affects hippocampus morphology and function [18,19].

Numerous neurons involved in the processing are endogenously activated during insomnia [20,21]. Their activity in the brain can be captured with implantable microelectrode arrays (MEA) [22,23]. The real-time detection of neuronal electrophysiological signals in vivo is important for discovering and understanding brain function mechanisms. Michigan electrodes are widely used to detect intracranial neurons with a high temporal and spatial resolution and capture the dynamic changes of individual neuron signals in real time [23]. In addition, with the development of chemical modification technology for the electrode surface, electrode impedance is significantly reduced after modification, and individual neuron activity can be detected more accurately [24,25].

We explored the effects of 5-HT deprivation-induced insomnia on neurons in brain regions, corresponding to memory and cognition. Since insomnia alters biological EEG, we hypothesized that the electrophysiological signals of the DRN and hippocampus of insomnia rats also altered. For this study, we design and fabricated an MEA to detect electrophysiological signals in the hippocampus and DRN simultaneously. The electrophysiological activities of the hippocampus and DRN in rats under normal, insomnia, and recovery conditions are detected in vivo using electrodes. Our purpose is to investigate the effects of insomnia induced by 5-HT on neurons in the hippocampus and DRN by analyzing the captured electrophysiological activity, and verifying whether the hypothesis is supported. Our research results provide new ideas for simultaneously detecting insomnia-related brain regions in multiple brain regions over long distances. It also promotes research on the intracerebral mechanism of insomnia caused by decreasing concentrations of 5-HT in the brain, affecting memory.

## 2. Materials and Methods

### 2.1. Reagents and Apparatus

The surface modification of the sites was performed using 3,4-Ethylenedioxythiophene (EDOT) (Aladdin, Shanghai, China), polystyrene sulfonate (PSS) (HEROCHEM, Shanghai, China), and short multi-walled carbon nanotubes (MWCNTs) (XFNANO, Nanjing, China). Reference 600 (Gamry Instruments, Warminster, PA, USA) evaluated the MEA site impedance. P-chlorophenylalanine (PCPA) (Sigma–Aldrich, Shanghai, China) and phosphate-buffered saline (PBS) (Sigma–Aldrich, Shanghai, China) were used to induce insomnia in rats. R520IP (RWD Life Science, Shenzhen, China) was used to anesthetize the rats. KeyGEN BioTECH (Nanjing, China) provided cell membrane orange fluorescent dye (DiI). Brain slices were manufactured by leica1950 (Leica Biosystems, Deer Park, IL, USA). The brain slices were imaged by ZEISS Axio Observer (ZEISS, Jena, Germany). The 128-channel neural data recording system (Blackrock Microsystems, Tampa, FL, USA), micropositioner (model 2662, David KOPF instrument, Los Angeles, CA, USA), and a classic stereotaxic instrument for rats (Stoelting, Wood Dale, IL, USA) were used to capture intracranial electrophysiological signals in rats.

### 2.2. Animal and Injection of P-Chlorophenylalanine

**Figure 1:**
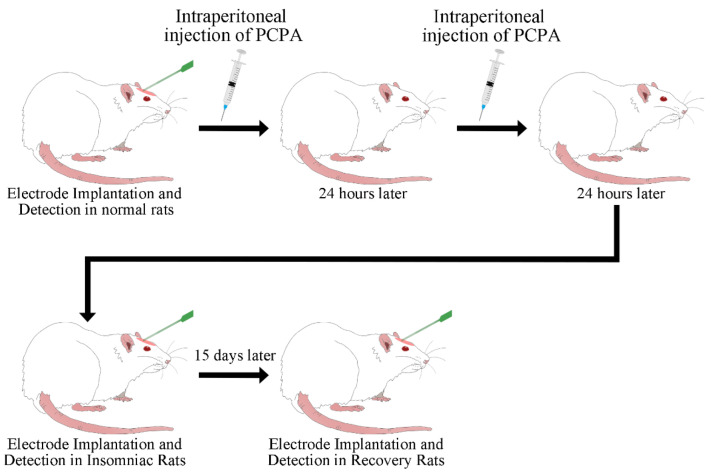
Schematic diagram of the experimental arrangement.

The schematic procedure of the experiment is shown in Figure 1. Twelve adult male Sprague Dawley (SD) rats weighing 250 g were randomly assigned to 3 groups: 4 rats were used as the control group, 4 rats were induced to become insomnia rats, and 4 recovered rats were detected 14 days after modeling. The laboratory temperature was kept at 25 °C to avoid the effects of low ambient temperatures [26].

P-chlorophenylalanine (PCPA) was recognized as a chemical agent to induce insomnia in rats [27,28,29], and it inhibited 5-HT synthesis [30]. PCPA was dissolved in PBS solution (30 mg/mL), and the solution was centrifuged for 30 min before injection. Rats were injected intraperitoneally with the solution (2.5 mL) on two consecutive days (24 h interval). Thirty hours after the first injection of PCPA, the rats lost their circadian rhythm and retained their insomnia, which verified the successful establishment of the model. All animal care and usage protocols were performed under the direction of institutional animal ethics committees.

### 2.3. Design and Modification of Microelectrode Arrays

**Figure 2:**
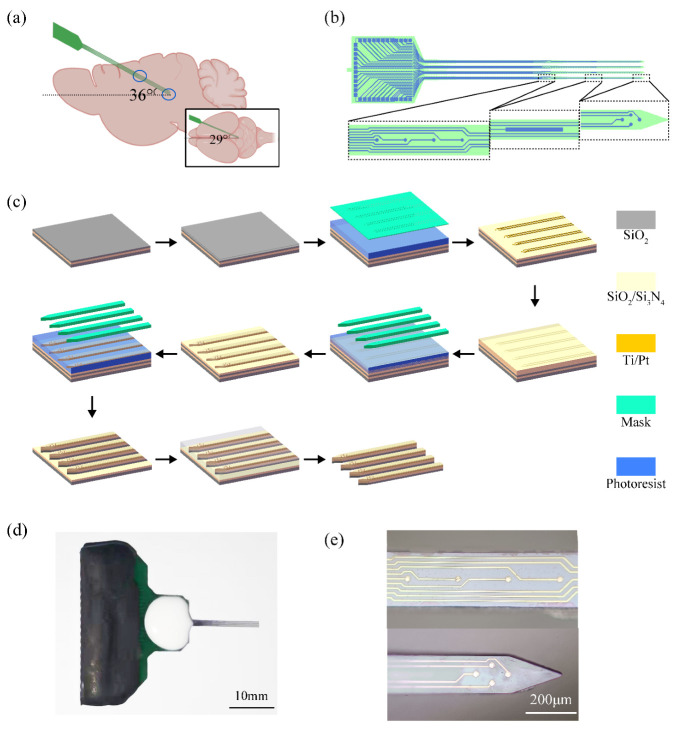
MEA design and fabrication. (**a**) MEA implantation path diagram in the sagittal plane. The blue circles are the location of the hippocampus and dorsal raphe nucleus. The smaller picture shows the MEA implantation path in the horizontal plane; (**b**) Site distribution map of the MEA; (**c**) The fabrication processes of the MEA. The processes are SOI wafer, thermal oxidation, photolithography, sputtering and stripping, plasma chemical vapor deposition, photolithography, oxygen plasma etching, photolithography, deep etching, black glue coating, and electrode release; (**d**) MEA finished product drawing, and (**e**) optical image of the MEA site. The upper image shows the site of the hippocampus, and the lower image shows the site of the dorsal raphe nucleus.

In this study, the MEA was implanted into the rat brain at an angle of 36° at the horizontal plane and 29° at the sagittal plane; the implantation depth was 11.8 mm (Figure 2a). The MEA consisted of four shanks, each with a U-shaped array of eight circular microelectrode sites (10 μm in diameter) (Figure 2b). The shank length was 12 mm, and the sites were distributed into two shank positions so that the rats’ hippocampus and DRN could be detected simultaneously. The site for detecting the hippocampus was 5.2 mm from the shank tip. There were sixteen sites in each of the two brain regions. An additional rectangular site was designed for each shank as the counter electrode. The MEA was implanted from the cortex of the rat brain (position coordinate, ML, 1.2 mm; AP, 0 mm; DV, −1.5 mm), through the hippocampus (position coordinate, ML, 0.5 mm; AP, 3.8 mm; DV, −4.9 mm), to the DRN (position coordinate, ML, 0 mm; AP, 6.4 mm; DV, −7.2 mm).

The MEAs were mass-fabricated in ultra-clean rooms by MEMS thin-film technology (Figure 2c): The SiO_2_ (500 nm) insulating layer grew by thermal oxidation on a silicon wafer on an SOI substrate; Ti/Pt (30 nm/250 nm) was sputtered and patterned using the lift-off process as a conductive layer; SiO_2_/Si_3_N_4_ (300 nm/500 nm) was deposited by plasma enhanced chemical vapor deposition as an insulating layer. The insulating layer on the surface of the MEA sites and bonding pads was removed by SF_6_ reactive ion etching. The probe shape was etched out using inductively coupled plasma reactive ion etching. The upper surface of the wafer was covered with pitch, and the lower surface was wet etched in a KOH solution (50%, 80 °C) until it stopped automatically; then, the buried oxide layer (2 μm) was removed in HF buffer solution. The wafer was placed in a developer solution to dissolve the pitch. Finally, the probes were soldered to the designed PCB and coated with silicone rubber (Figure 2d).

The naked sites are shown in Figure 2e. The purpose of surface modification of MEA sites was to increase the site’s surface area. In addition, for the same tube diameter, the shorter the tube length, the larger the specific surface area of the multi-walled carbon nanotubes [31]. Therefore, we adopted PtNPs/short MWCNT-PEDOT: PSS as the modification scheme for this study. A three-electrode setup was used in this experiment (MEA: working electrode; Pt: counter electrode; Ag|AgCl: reference electrode). First, platinum nanoparticles (PtNPs) were electrodeposited onto sites by chronoamperometry (CA, −1.0 V, 40 s) in a mixed solution of 48 mM chloroplatinic acid and 4.2 mM lead acetate. Then, the electrodeposition of short-MWCNT-PEDOT: PSS was performed by cyclic voltammetry sweeping (0–0.9 V) for 10 cycles in the mixture solution of PSS (0.2 M), EDOT (40 mM), and short-MWCNTs (4 mg/mL).

To explore the electrochemical performance of PtNPs/short MWCNT-PEDOT: PSS-modified MEA sites, the redox potentials of MEA sites modified with PtNPs/MWCNT-PEDOT: PSS or PtNPs/short MWCNT-PEDOT: PSS were measured in the solution contained dopamine (DA) (100 μM) and 5-HT (100 μM) via cyclic voltammetry. The minimum detection limit and linear calibration curve were measured by chronoamperometry (0.495 V) in a PBS solution after the multiple addition of 5-HT solution (10 μL) at different concentrations. The Nafion layer was coated on the MEA site, which blocked the interference of anions in the brain. The selectivity was measured by chronoamperometry (0.495 V) in PBS solution after adding 5-HT solution, DA solution, uric acid solution (UA), ascorbic acid solution (AA), and 3,4-dihydroxyphenylacetic acid solution (DOPAC) in sequence.

### 2.4. Surgical Procedures and Implant Pathway Check

Rats were anesthetized by isoflurane and fixed in a stereotaxic frame during the experiment. The craniotomy was conducted at the coordinates of 1.5 mm posterior to bregma and 1.2 mm lateral from the midline. Another site was drilled into the skull’s surface, and a stainless-steel needle was fixed there as the ground electrode.

After the collection of electrophysiological signals, the brain was dehydrated and sectioned, and the DiI marked the wound trace. Sections with coordinates of 1.5, 4.9, and 7.2 mm posterior to bregma were viewed through a microscope. Sections of coordinates 1.5 mm after bregma were observed by a microscope and compared to the rat brain atlas to verify whether the electrode implantation was exactly at the predetermined position.

### 2.5. δ Wave Slope Analysis

According to the classification of brain waves, the LFP’s power was divided into four bands: δ (0–4 Hz), θ (4–7 Hz), α (8–12 Hz) and β (13–30 Hz) [32]. The steps to extracting the slope of the δ wave [33]: (1) the signal was filtered into a δ wave by low-pass filtering (4 Hz); (2) the zero-crossing point was determined; (3) the extreme values on both sides of the zero-crossing point was determined; (4) the first segment slope of the δ wave was the slope of the positive deflection through the zero-crossing point, and the second segment slope of the δ wave was the slope of the negative deflection through the zero-crossing point; (5) the sum of the durations of adjacent positive and negative deflections should be longer than 0.25 s; and (6) the slopes of the two segments were averaged and counted.

### 2.6. Data Acquisition and Statistics

The stereotaxic frame with the rat was placed into a grounded and shielded box during the measurement to reduce external interference. The MEA was connected to a 128-channel neural data recording system to simultaneously detect electrophysiological signals from neurons in the DRN and hippocampus of rats. The sampling frequency was set to 30 kHz to capture neurophysiological signals in real time. Then, the local field potential (LFP) (low-pass filter of 250 Hz) and spike (high-pass filter of 250 Hz) were extracted from the data. After recording the measurements, NeuroExplorer Version 4 (Nex Technologies, Colorado Springs, CO, USA) was used to analyze the data. Statistical analyses and graphs were performed with OriginPro 2021 (OriginLab Corporation, Northampton, MA, USA). Data were calculated as the mean ± SEM. A two-sample analysis was used. A statistical significance of *p* < 0.05 was set for all analyses.

## 3. Results

### 3.1. Sensor Evaluation

**Figure 3:**
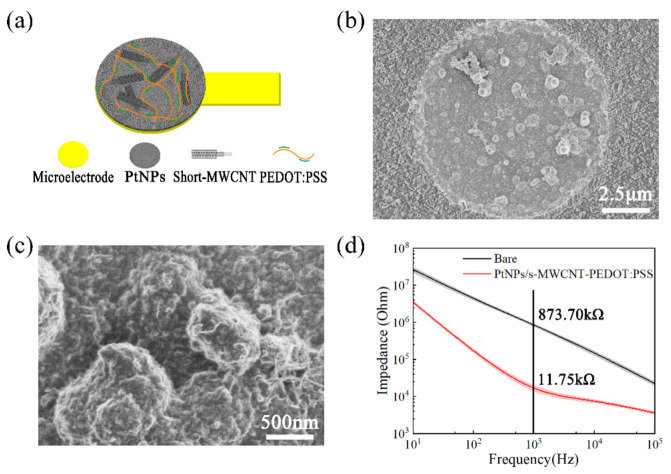
Morphology and impedance characteristics of the MEA sites. (**a**) MEA site modification protocol; (**b**) Scanning electron microscopy images (magnification: 5 k) of the PtNPs/short MWCNT-PEDOT: PSS-modified MEA sites; (**c**) Scanning electron microscopy images (magnification: 30 k) of the PtNPs/short MWCNT-PEDOT: PSS-modified MEA sites; (**d**) The EIS was recorded in a frequency range from 1 Hz to 1 MHz with a sinusoidal potential of 20 mV peak–peak amplitude. The mean impedance of the MEA decreased from 873.70 ± 44.20 kΩ to 11.75 ± 2.30 kΩ (*n* = 32) at 1 kHz after the PtNPs/short MWCNT-PEDOT: PSS modification.

The modification scheme of PtNPs/short MWCNT-PEDOT: PSS is shown in Figure 3a. After modification, the surface area of the MEA site significantly increased (Figure 3b,c). The electrochemical impedance spectrum (EIS) is an effective and basic electro-chemical cell analysis method used to characterize the sensor impedance. Figure 3d details the impedance of the MEA sites at 1 kHz before and after the PtNPs/short MWCNT-PEDOT: PSS modification. Before modification, the average impedance of 32 sites was 873.70 ± 44.20 kΩ. After modification, the impedance decreased to 11.75 ± 2.30 kΩ, significantly reducing to 74.36 times smaller.

**Figure 4:**
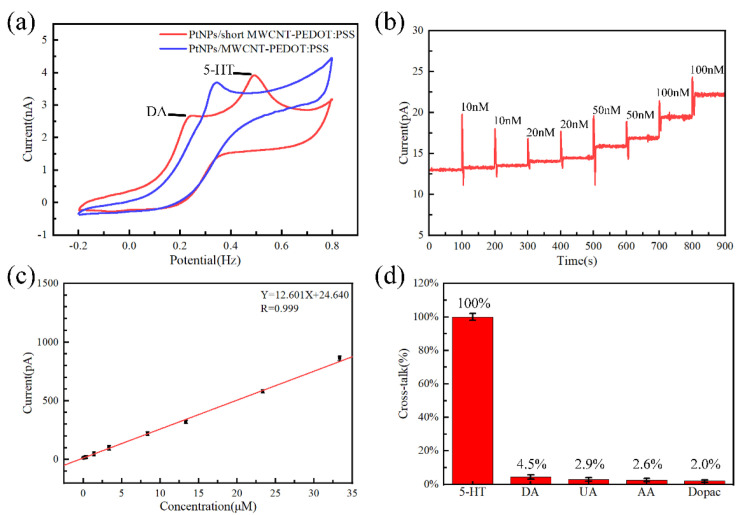
Electrochemical performance of the MEA. (**a**) The cyclic voltammetry of the MEA sites modified with PtNPs/MWCNT-PEDOT: PSS or PtNPs/short MWCNT-PEDOT: PSS in the solution contained DA (100 μM) and 5-HT (100 μM); (**b**) Minimum detection limit of the MEA tested at tiny concentrations of 5-HT solution; (**c**) The fitting curve between the 5-HT response current and the corresponding concentration; (**d**) Selective results of the 5-HT microelectrode arrays against common interferences. DA: dopamine; UA: uric acid; AA: ascorbic acid; DOPAC: 3,4-dihydroxyphenylacetic acid.

The cyclic voltammetry of the MEA sites modified with PtNPs/MWCNT-PEDOT: PSS or PtNPs/short MWCNT-PEDOT: PSS is shown in Figure 4a. The redox curve of the MEA site modified with PtNPs/MWCNT-PEDOT: PSS had only one oxidation peak (0.350 V), which cannot distinguish DA and 5-HT in solution. The redox curve of the PtNPs/short MWCNT-PEDOT: PSS-modified MEA site has two oxidation peaks, which can clearly distinguish DA (0.250 V) and 5-HT (0.495 V) in the solution. When a 50 nM solution of 5-HT was dropped in the PBS, the current changed significantly (signal-to-noise ratio > 3), so the minimum detection limit of 5-HT for this MEA was 50 nM (Figure 4b). In addition, the 5-HT solution at a concentration of 10 nM–10 uM was sequentially dropped into the PBS solution. When the linear range was 10 nM–35 μM, the sensitivity of MEA was 12.601 pA/μM (R = 0.999) (Figure 4c). Finally, MEA has excellent selectivity (Figure 4d). The selectivity ratios of MEA to DOPAC, Glu, UA and AA were 95.5, 97.1, 97.5 and 98.0%, respectively.

### 3.2. MEA Implantation Path

**Figure 5:**
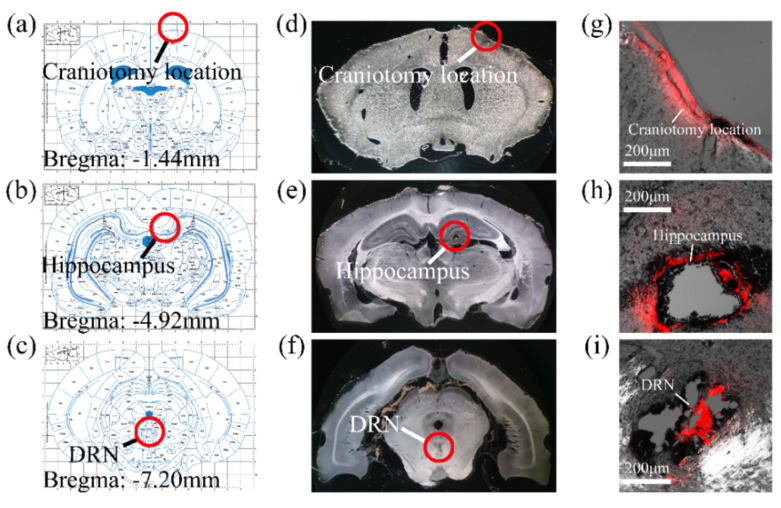
MEA implantation path verification. (**a**) Craniotomy location in the brain atlas; (**b**) Hippocampus in the brain atlas; (**c**) DRN in the brain atlas; (**d**) Craniotomy location in the brain slices; (**e**) Hippocampus in the brain slices; (**f**) DRN in the brain slices; (**g**) DiI traces at the craniotomy location by fluorescence microscopy; (**h**) DiI traces at the hippocampus by fluorescence microscopy; and (**i**) DiI traces in the DRN by fluorescence microscopy. (**a**–**i**) correspond with the coronal plane.

The MEA implantation path was more clearly determined using the staining microscope. According to the stained area of the brain slice, it was found that the MEA reached the DRN from the craniotomy position through the hippocampus. The pathways did not shift from the expected positions after being compared to the brain atlas (Figure 5).

### 3.3. Analysis of Neural Spikes and Local Field Potentials

**Figure 6:**
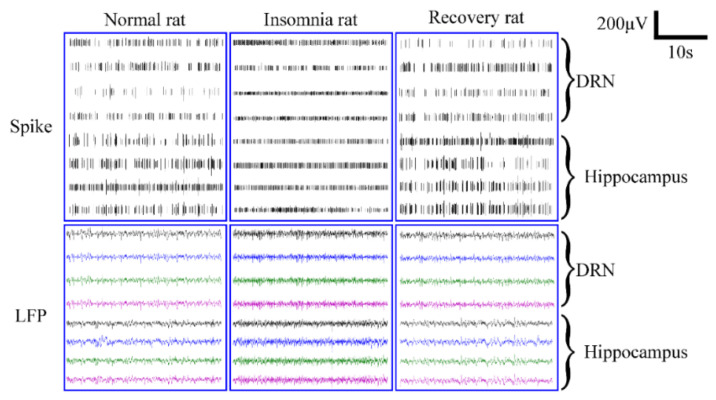
Captures of neural spiking activity (upper panel) and local field potentials (LFPs) (lower panel) across eight channels of rats in normal, insomnia, and recovery states.

MEA captured the electrophysiological signals of normal, insomnia, and recovery rats, and spikes of over eight channels and were successfully recorded at a signal-to-noise ratio of above 3.5 (Figure 6). The MEA sites were in close contact with neurons in brain regions, so the information displayed by each channel clearly reflected neural information in the hippocampus and DRN. The real-time neural spikes and local field potentials (LFPs) were recorded simultaneously in eight different channels over a 30 s period. As seen in Figure 6, spikes in both brain regions of normal rats fired at a relatively slow rate. Significantly, spike discharges in insomniac rats increased and became fiercer, accompanied by a frequent fluctuation of LFP.

**Figure 7:**
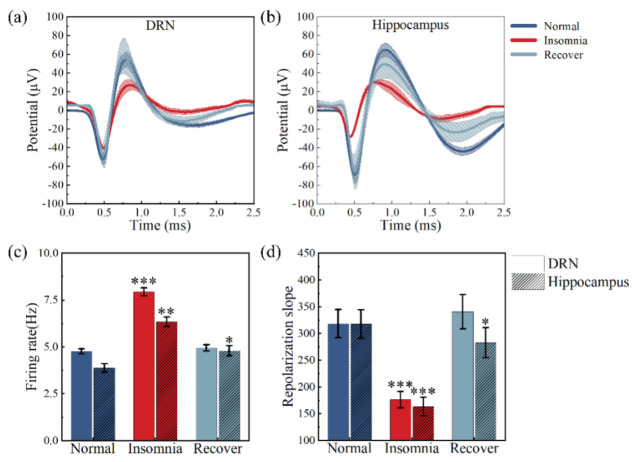
Statistics for spike waveform and firing rate of the rats’ dorsal raphe nucleus (DRN) and hippocampus in normal, insomnia and recovery states (*n* = 12). (**a**) Spike waveform statistics in the DRN; (**b**) Spike waveform statistics in the hippocampus; (**c**) Statistics for neural firing rate in the DRN and hippocampus; (**d**) Repolarization slope of spikes in the DRN and hippocampus. (* *p* < 0.05, ** *p* < 0.01, *** *p* < 0.001, compared to normal in the same brain region).

Next, the spikes were analyzed in detail. According to Figure 7a,b, the spike amplitude (64.63 μV, 57.00 μV) in the DRN and hippocampus of insomnia rats was significantly smaller than normal (108.00 μV, 135.45 μV) and recovery rats (102.17 μV, 110.25 μV). In addition, the firing rates of insomnia rats were consistently higher than those of normal and recovery rats. After 14 days, spikes and LFPs were restored in both brain regions of the recovery rats. We counted the spike’s firing rate and found that the hippocampal spike’s firing rate of insomnia rats increased from 3.88 to 6.34 Hz after modeling, then dropped to 4.80 Hz after 14 days. Similarly, insomnia rats’ DRN spike’s firing rate increased from 4.76 to 7.94 Hz after modeling, then dropped to 4.95 Hz after 14 days (Figure 7c).

The spikes’ action potential was divided into three parts: depolarization, repolarization, and hyperpolarization. The spike duration in this study was defined as the time of repolarization. The DRN spike duration did not change significantly, and the spike durations were 0.33, 0.36, and 0.30 s in normal, insomnia, and recovery rats, respectively. The hippocampal spike duration (0.34 s) in insomnia rats was much smaller than normal (0.40 s) and recovered rats (0.39 s). The repolarization slopes of the DRN spikes in the three states were 318.18 ± 26.39, 176.16 ± 15.41, and 340.56 ± 32.02. The repolarization slopes of the hippocampal spikes in the three states were 317.50 ± 26.83, 163.24 ± 17.5, and 282.69 ± 27.98 (Figure 7d).

### 3.4. Analysis of Powers

**Figure 8:**
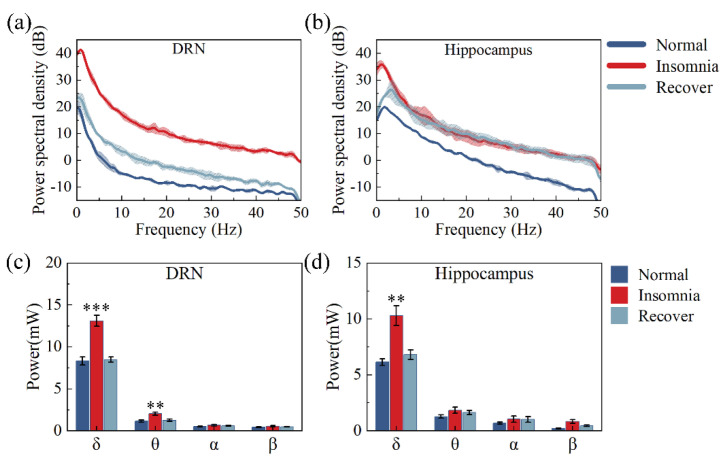
Statistics for PSD and power of rats’ dorsal raphe nucleus (DRN) and hippocampus of rats in normal, insomnia and recovery states (*n* = 12). (**a**) PSD statistics in DRN; (**b**) PSD statistics in hippocampus; (**c**) Power statistics in the DRN; (**d**) Power statistics in the hippocampus. δ: 0–4 Hz; θ: 4–7 Hz; α: 8–12 Hz; β: 13–30 Hz. (** *p* < 0.01, *** *p* < 0.001, compared to normal in the same brain region).

The power spectral density (PSD) of the LFP’s hippocampus and DRN power were mainly distributed in the lower frequency band; the power significantly increased after the insomnia model (Figure 8a,b). We have more refined statistics at the power of 0–30 Hz. The δ wave power of the hippocampus and DRN increased by 67.75 and 57.57% after insomnia modeling. After 14 days of insomnia modeling, the δ wave power of the hippocampus and DRN were only 10.91 and 4.95% higher than in normal rats. Meanwhile, the power changes in other frequency bands were not significant (Figure 8c,d).

### 3.5. δ Wave Slope Analysis

**Figure 9:**
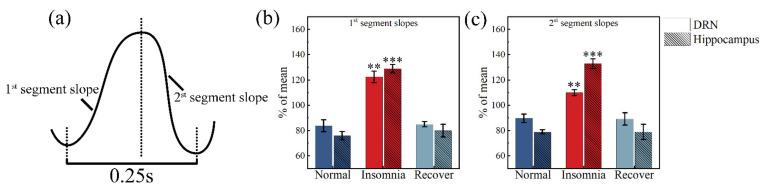
Calculations and analysis of δ wave slope in rats’ dorsal raphe nucleus (DRN) and hippocampus in normal, insomnia and recovery states (*n* = 12). (**a**) Representative individual LFP δ wave; (**b**) first segment slopes in the DRN and hippocampus. (**c**) Second segment slopes in the DRN and hippocampus. (** *p* < 0.01, *** *p* < 0.001, compared to normal in the same segment).

The δ wave slope is an LFP component closely related to sleep [33,34]. Representative individual LFP delta waves were selected through our algorithm (Figure 9a).

By calculating the slopes, the first and second segment slopes in the DRN increased from 83.72, 89.63 to 122.41, 109.91 after insomnia modeling (Figure 9b). The first and second segment slopes in the hippocampus increased from 76.01, 78.94 to 128.85, 132.93 after insomnia modeling (Figure 9c).

### 3.6. Analysis between Firing Rate and Local Field Potential

The spike’s firing rate increased in the hippocampus and DRN of insomnia rats. Simultaneously, the δ wave power in the hippocampus and DRN of insomnia rats also increased. To determine whether there was a link between power and spike, we fitted the relationship between neuronal firing rate and δ wave power in the DRN and hippocampus.

**Figure 10:**
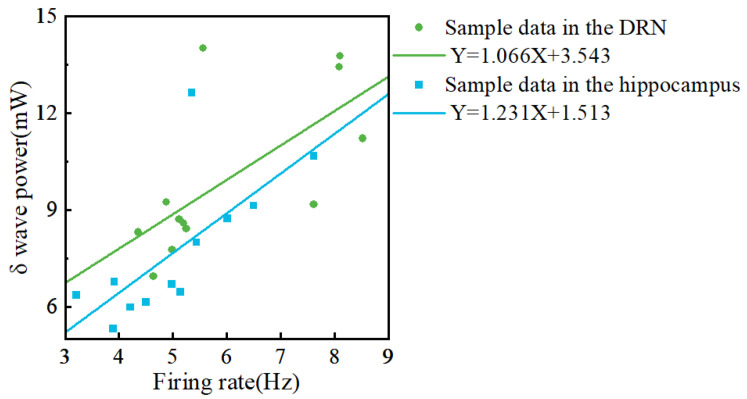
The relationship between neuronal firing rate and δ wave power in the dorsal raphe nucleus (DRN) and hippocampus. X: Firing rate; Y: δ wave power. The green line is the fitted line for the DRN data. The blue line is the fitted line for the hippocampal data.

Our results showed a positive proportional relationship between the firing rate and δ wave power. As shown in Figure 10, the slope of the fitted line was 1.066 for the DRN, whereas the slope of the fitted line was 1.231 for the hippocampus.

## 4. Discussion

In order to investigate the neural mechanisms associated with insomnia, we designed a PtNPs/short MWCNT-PEDOT: PSS-modified MEA for simultaneously detecting firing patterns in the hippocampus and DRN of rats. Twelve rats were divided equally into three groups: normal, insomnia, and recovery. The designed MEA was implanted into the DRN through the hippocampus at a set angle. We further explored electrophysiological signal changes in the hippocampus and DRN of rats under three conditions: normal, insomnia, and recovery. Our results showed that the spike amplitude in the hippocampus and DRN of PCPA-induced insomnia rats was significantly reduced, whereas the spiking rate was significantly increased. In addition, the 0–50 Hz power of the rats after the insomnia model was significantly increased, and the main change was in the δ band. The firing rate of the DRN and the hippocampus was also positive proportional to the δ wave power. Our findings suggested that implanting MEA into the brain at specific angles simultaneously detects multiple deep brain regions, and that 5-HT-induced insomnia altered neuronal firing patterns in the hippocampus and DRN.

The shanks of the MEA were 12 mm long. It could not only detect superficial regions, such as the cerebral cortex or hippocampus, but also had the ability to detect deep brain regions, such as the DRN. Meanwhile, the MEA sites were only 10 μm in diameter so that the activity of individual neurons was detectable [35]. In this study, the impedance of the MEA sites was reduced to 11.75 ± 2.30 kΩ by modifying PtNPs/short MWCNT-PEDOT: PSS. The impedance of the modified MEA site was only 1.34% of that of the bare MEA site, which was superior to our previous design [36]. In this way, the MEA could clearly capture the neuronal spikes in the DRN and hippocampus of insomnia rats in vivo. In addition, the PtNPs/short MWCNT-PEDOT: PSS-modified MEA possessed the ability to detect 5-HT and DA simultaneously due to the better electrochemical performance of short MWCNTs [31]. The molecular structure of DA was very similar to 5-HT, and DA could affect the current response of 5-HT. Our designed MEA had different oxidation potentials for 5-HT and DA, which could exclude the interference of DA.

In this study, when 5-HT-deficient rats suffered from insomnia, spike discharges in the DRN and hippocampus were more frequent, and LFP fluctuations were more intense (Figure 6). Many 5-HT neurons were present in the DRN, and the hippocampus was also a brain region with efferent connections to 5-HT neurons [14]. Previous studies have shown that 5-HT is a class of inhibitory neurons [30]. The rat brain was depleted of 5-HT, so inhibited neuronal activity in the DRN and hippocampus increased significantly (Figure 6). As a result, there was a significant increase in the spike firing rate on a microscopic level. Our study also showed that, after insomnia in rats, the firing rate change (66.81%) of the DRN was higher than (63.40%) the hippocampus; this may be due to the denser distribution of 5-HT_1A_ neurons in the DRN than in the hippocampus.

A detailed analysis of spike waveforms revealed that, after insomnia modeling, the slope of the spike depolarization did not change (Figure 7a,b), indicating that sodium currents in the DRN and hippocampus are associated with cellular electrical signaling were not affected. However, the spike repolarization slope was significantly reduced after insomnia modeling, possibly due to the lack of 5-HT activation for potassium currents [37]. In addition, the spike duration in hippocampal neurons was shortened due to increased calcium currents in hippocampal neurons [38]. The same phenomenon was found in a study on the Aplysia nervous system [39], which included 5-HT-induced widening of spikes in sensory neurons.

The amplitudes of the spike depolarization were significantly reduced in both brain regions, possibly due to the combined effect of changes in calcium and potassium currents (Figure 7d). Likewise, 5-HT may amplify fast extensor tibiae motor neuron spikes by modulating the potassium conductance responsible for the spike repolarization, similar to the results of this study [40]. In addition, after insomnia in rats, the amplitude change (57.92%) of the hippocampus was higher (40.16%) than in the DRN (Figure 7). This phenomenon suggested that the potassium conductance of neurons in the dorsal raphe nucleus was more affected by 5-HT than hippocampal neurons. Altogether, the firing rate of DRN’s spike and the hippocampal spike’s amplitude more effectively reflected the sleep situation of 5-HT-deficient rats.

In this study, after insomnia modeling, increased power in the 0–50 Hz of the LFP (Figure 8a,b) and individual sub-bands within each were observed from multiple channels of the MEA, suggesting that PCPA-induced insomnia affected groups of neurons’ activity. In addition, the increase in power mainly depended on the increase in δ wave power (Figure 8c,d). The δ oscillation between regular frequency and large amplitude played an important role in sleep. There was a marked increase in δ power when rats were not asleep for extended periods, consistent with the present study [41,42]. Sleep studies in animals surfaced that EEG δ power during non-rapid-eye-movement sleep increases proportionally with wake duration [43,44]. In our study, after insomnia modeling, the change in δ power in the hippocampus was larger than in the DRN, indicating that hippocampal δ power reflected insomnia caused by 5-HT deficiency more effectively. Furthermore, our study found that the two-segment slopes of the δ wave in insomnia rats increased (Figure 9b,c), indicating that they could be used as two parameters to reflect the sleep state of rats [33]. Moreover, changes to the two-segment slopes in the hippocampus (41.01%, 40.62%) were more effective in reflecting insomnia than changes in the DRN’s two-segment slopes (31.61%, 18.45%).

The spike reflected the electrophysiological activity of a single neuron, and the LFP reflected the electrophysiological activity of a group of neurons in the vicinity of the site. As shown in Figure 10, increased firing rates of individual neurons led to increased δ power in the DRN and hippocampus of insomnia rats, consistent with our previous study [36]. Studies have shown that the LFP power is proportional to the spike firing rate [45]. Similarly, our study found that the change in δ power in the hippocampus was larger than in the DRN for the same difference in firing rate. This finding might indicate that neurons in different brain regions contribute differently to δ wave power. Much of the relationship between δ power and sleep has been validated by EEG signals; however, studies using EEG δ power alone to measure sleep status remain sparse. The relationship between intracranial δ power and sleep quality indicates the possibility of using δ power in multiple brain regions to measure sleep quality.

Finally, we also found that the amplitude, firing rate, and repolarization slope of the recovery rats’ hippocampus were significantly different from the normal rats’ hippocampus. Studies have shown that insomnia clouds significantly affect the memory function of rats [15,46,47]. The abnormal firing pattern of neurons in the hippocampus may involve compensatory brain activation to maintain memory and cognition [48]. Therefore, the negative effects of long-term insomnia on memory may be difficult to recover from quickly or completely [49,50].

## 5. Conclusions

Essentially, an MEA was designed to detect the hippocampus and DRN of rats simultaneously. Electrophysiological signals from two brain regions of normal, insomnia, and recovery rats were captured in vivo through designed and modified MEA. Our preliminary findings showed that insomnia rats have more intensive spikes and severe LFP fluctuations. Microscopically, after insomnia, the spike amplitudes decreased, cell repolarization slope decreased, and the spike firing rate increased in the DRN and hippocampus. Then, δ wave power increased in insomnia rats. In addition, two segment slopes of the δ wave in the DRN and hippocampus also increased in insomnia rats. Finally, we found that the firing rates of the DRN and hippocampus were positive in relation to the δ wave power. Our research lays the foundation for the simultaneous electrophysiological signal detection of deep multi-brain regions in vivo and preliminarily explores changes in the neuronal firing patterns of insomnia-induced rats by 5-HT deficiency. Unfortunately, our study did not combine cytology with the exploration of the causes of neuronal electrophysiological signal changes in depth. We can combine chemical or electrical stimulation to achieve high-efficiency treatment for insomnia in the future.

## Data Availability

Data are contained within the article.
